# 15d-PGJ_2_-loaded nanocapsules ameliorate experimental gout arthritis by reducing pain and inflammation in a PPAR-gamma-sensitive manner in mice

**DOI:** 10.1038/s41598-018-32334-0

**Published:** 2018-09-18

**Authors:** Kenji W. Ruiz-Miyazawa, Larissa Staurengo-Ferrari, Felipe A. Pinho-Ribeiro, Victor Fattori, Tiago H. Zaninelli, Stephanie Badaro-Garcia, Sergio M. Borghi, Ketlem C. Andrade, Juliana T. Clemente-Napimoga, Jose C. Alves-Filho, Thiago M. Cunha, Leonardo F. Fraceto, Fernando Q. Cunha, Marcelo H. Napimoga, Rubia Casagrande, Waldiceu A. Verri

**Affiliations:** 1 Departamento de Ciências Patológicas, Universidade Estadual de Londrina-UEL, Rod. Celso Garcia Cid, Km 380, PR445, 86057-970, Cx. Postal 10.011 Londrina, Paraná Brazil; 2 Laboratory of Immunology and Molecular Biology, São Leopoldo Mandic Institute and Researcher Center, Campinas, Brazil; 3 Department of Pharmacology, Ribeirão Preto Medical School, University of São Paulo, Avenida Bandeirantes s/n, 14050-490 Ribeirão Preto, São Paulo Brazil; 4 Department of Environmental Engineering, São Paulo State University, Sorocaba, Brazil; 5 Departamento de Ciências Farmacêuticas, Universidade Estadual de Londrina-UEL, Avenida Robert Koch, 60, Hospital Universitário, 86038-350 Londrina, Paraná Brazil

## Abstract

Gout arthritis (GA) is a painful inflammatory disease in response to monosodium urate (MSU) crystals in the joints. 15deoxy-Δ^12,14^-prostaglandin J_2_ (15d-PGJ_2_) is a natural activator of PPAR-γ with analgesic, anti-inflammatory, and pro-resolution properties. Thus, we aimed to evaluate the effect and mechanisms of action of 15d-PGJ_2_ nanocapsules (NC) in the model of GA in mice, since a reduction of 33-fold in the dose of 15d-PGJ_2_ has been reported. Mice were treated with 15d-PGJ_2_-loaded NC, inert NC, free 15d-PGJ_2_ (without NC), or 15d-PGJ_2_-loaded NC+ GW9662, a PPAR-γ inhibitor. We show that 15d-PGJ_2_-loaded NC provided analgesic effect in a dose that the free 15d-PGJ_2_ failed to inhibiting pain and inflammation. Hence, 15d-PGJ_2_-loaded NC reduced MSU-induced IL-1β, TNF-α, IL-6, IL-17, and IL-33 release and oxidative stress. Also, 15d-PGJ_2_-loaded NC decreased the maturation of IL-1β in LPS-primed BMDM triggered by MSU. Further, 15d-PGJ_2_-loaded NC decreased the expression of the components of the inflammasome *Nlrp3*, *Asc*, and *Pro-caspase-1*, as consequence of inhibiting NF-κB activation. All effects were PPAR-γ-sensitive. Therefore, we demonstrated that 15d-PGJ_2_-loaded NC present analgesic and anti-inflammatory properties in a PPAR-γ-dependent manner inhibiting IL-1β release and NF-κB activation in GA. Concluding, 15d-PGJ_2_-loaded NC ameliorates MSU-induced GA in a PPAR-γ-sensitive manner.

## Introduction

Over the past decade, the concept involving the resolution of the acute inflammatory process has changed. Once thought to be a passive process, the resolution of inflammation is now understood as a process tightly regulated by pro-resolving mediators, which include the omega-3 fatty acid-derived molecules, so-called specialized pro-resolving lipid mediators (SPMs)^1^. Several other isolated SPMs such as RvE1, RvD1, MaR1, and LxA_4_ reduce pain by inhibiting the activity of nociceptor neurons or by reducing inflammation^1,2^. Thus, these data indicate that pro-resolving molecules present analgesic and anti-inflammatory properties. Peripheral mechanisms of SPMs are mainly related to reducing neutrophil counts and NF-κB activation^1,2^.

Gout arthritis is a painful inflammatory disease in response to monosodium urate (MSU) crystals in the joints^3,4^. MSU crystals induce the production of IL-1β in an NLRP3-dependent manner^4–6^. This is the main step in the pathogenesis of the disease and pain experienced by the patients (namely acute flares)^4,6^. Patients seek medical care due to acute flares^3^. In spite of self-resolving (about 10 days), gout acute flares are one of the most painful experiences to humans^3^. The management of pain in gout acute flares depends on steroidal and non-steroidal anti-inflammatory drugs (NSAIDs), colchicine and biological agents targeting IL-1^3^. However, the use of these drugs lack safety in patients with comorbidities (NSAIDs), often cause severe side effects (NSAIDs, colchicine, and corticoids), present high cost (biological agents), or possess non-satisfactory analgesic effects in some patients with gout^3,4^. Thus, novel analgesic drugs are still needed.

The cyclopentatone type-15deoxy-Δ^12,14^-prostaglandin J_2_ (15d-PGJ_2_) is formed as a consequence of dehydration of prostaglandin D_2_ and is a natural ligand that activates the peroxisome proliferator-activated receptor gamma (PPAR-γ)^7,8^. 15d-PGJ_2_ is reported to stimulate transcription of several target genes *via* PPAR-γ-dependent mechanisms and to promote the resolution of inflammatory process as an endogenous feedback regulator of the inflammatory process *in vivo*^7,9^. Moreover, 15d-PGJ_2_ has been reported to reduce NF-κB activation, an effect intermediated by the activation of PPAR-γ (an indirect mechanism)^10^ or by targeting IκB kinase (IKK) or p65 NF-κB subunit (a direct mechanism)^11,12^. In addition to resolve inflammation, activation of PPAR-γ by other molecules also induces analgesia^13,14^, which indicates that activating this nuclear receptor represents a promising analgesic approach. In fact, treatment with 15d-PGJ_2_ reduces temporomandibular joint pain^15–18^. Interestingly, nanoencapsulated 15d-PGJ_2_ requires a dose 33 times lower to inhibit inflammation^19^ and pain compared to the effects of free 15d-PGJ_2_ (30 μg/kg *vs*. 1000 μg/kg)^15^. Nanocapsules consist of polymeric involucres around a nucleus generally oily used to prolong pharmacological activity and decrease toxicity of molecules^20^. 15d-PGJ_2_ was shown to be released slowly from nanocapsules, which avoided inactivation or reduction of its biological activity^21,22^ by reactions that could include Michael’s addition^23^. Another possibility is that cells could uptake nanoparticles loaded with 15d-PGJ_2_ resulting in enhanced 15d-PGJ_2_ levels inside the cells as well as increased pharmacological response. Thus, this enhancement of 15d-PGJ_2_ activity demonstrates that the nanoencapsulation process worked properly to increase the efficacy of 15d-PGJ_2_^18^. In fact, other alternatives, such as topical delivery of 15d-PGJ_2_ further corroborates that changes in the formulation and delivery of this molecule increases its activity, *i.e*., it is required a lower dose to achieve the same effect compared to simply dissolved 15d-PGJ_2_^16^. Thus, in this work, our aim was to investigate the efficacy and mechanisms of action of 15d-PGJ_2_-loaded nanocapsules (NC) in MSU-induced inflammation and pain in mice.

## Results

### 15d-PGJ_2_-loaded nanocapsules (NC) inhibit MSU-induced mechanical hyperalgesia and joint swelling via PPAR-γ

**Figure 1:**
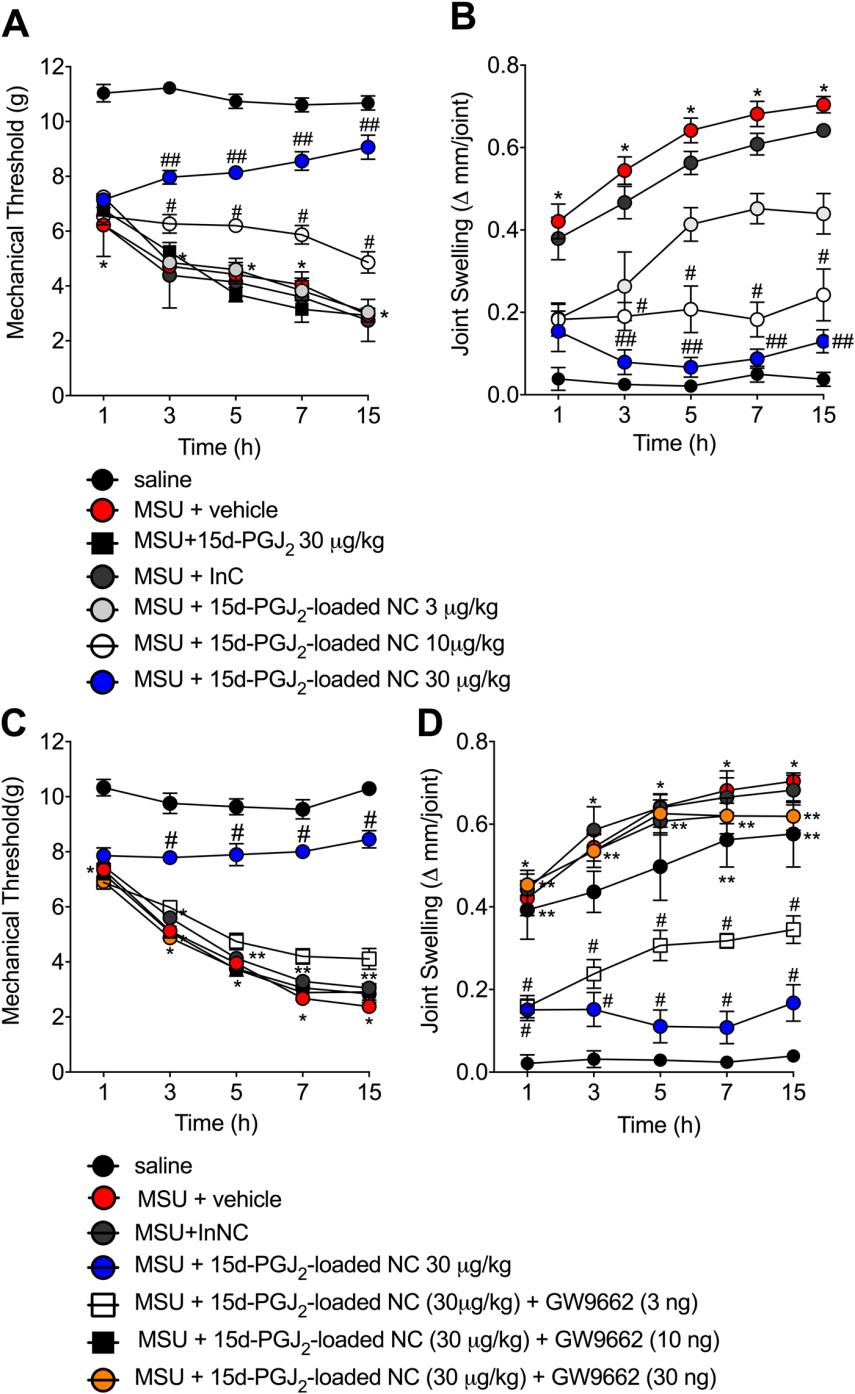
15d-PGJ_2_-loaded NC inhibit the pain-like behavior triggered by mechanical stimulus and joint swelling induced by MSU. Mechanical hyperalgesia (**A**,**C**) and joint swelling (**B**,**D**) were evaluated 1, 3, 5, 7 and 15 h after MSU injection. Results are mean ± SEM, n = 6 mice per group in each experiment, two independent experiments (*p < 0.05 vs. control group; ^#^p < 0.05 vs. vehicle mg/kg group, ^##^p < 0.05 vs. 15d-PGJ_2_-loaded NC 10 μg/kg group. Two-way ANOVA and post-test of Tukey).

First, it was addressed whether or not 15d-PGJ_2_-loaded NC (s.c., 30 min before MSU injection, 3–30 μg/kg) could reduce mechanical hyperalgesia and joint swelling induced by MSU crystals. MSU injection induced mechanical hyperalgesia (Fig. 1A) and joint swelling (Fig. 1B) at all measured time points. 15d-PGJ_2_-loaded NC at 10 μg/kg reduced MSU-induced mechanical hyperalgesia (Fig. 1A) and joint swelling (Fig. 1B) (3–15 h). The dose of 30 µg/kg of 15d-PGJ_2_-loaded NC was able to inhibit these same parameters with statistical difference when compared to the dose of 10 μg/kg. Of note, all three doses of 15d-PGJ_2_-loaded NC reduced joint swelling at 1 h after MSU injection. However, the lower dose (3 μg/kg) lost effect over time, the mid dose (10 μg/kg) maintained its inhibitory effect not allowing the increase of joint swelling, and the higher dose (30 μg/kg) presented an increase of effect over time. It is possible that the lower dose of 15d-PGJ_2_-loaded NC was sufficient to inhibit MSU-induced joint swelling at 1 h, but since there was an increase of swelling as the inflammation developed, only higher doses were enough to limit swelleing progression. The inert nanocapsules (InNC), *i.e*. the nanocapsules without 15d-PGJ_2_, showed no effect. Thus, the dose of 30 µg/kg, s.c of 15d-PGJ_2_-loaded NC was select for the next experiments.

As 15d-PGJ_2_ is an endogenous regulator of PPAR-γ^7^ and activation of this ligand-activated transcription factor has been shown to have analgesic properties^15,24^, we next evaluated whether the effect of 15d-PGJ_2_-loaded NC occurred in a PPAR-γ-sensitive manner. To this end, it was used GW9662 (i.art., 30 min before MSU injection, 3–30 ng per joint), a selective and irreversible antagonist of PPAR-γ^25^. Treatment with 15d-PGJ_2_-loaded NC at 30 μg/kg reduced the pain-like behavior triggered by mechanical stimulus and joint swelling induced by MSU crystals. Both mechanical hyperalgesia (15 h, Fig. 1C) and joint swelling (15 h Fig. 1D) were reverted by the treatment with GW9662 at 10 and 30 ng without difference between these two doses. GW9662 at 3 ng was unable to reduce the analgesic effect of 15d-PGJ_2_-loaded NC at 30 μg/kg (Fig. 1C). Thus, dose 10 ng of GW9662 was select for the next experiments to further investigated the involvement of PPAR-γ in this model. It was also used free 15d-PGJ_2_, *i.e*., without nanocapsule, at 30 μg/kg (the same dose of 15d-PGJ_2_-loaded NC, s.c. 30 min before MSU injection) to investigate whether nanoencapsulation of 15d-PGJ_2_ enhances the analgesic effect of this molecule. Neither free 15d-PGJ_2_ (without nanocapsules) nor inert nanocapsules reduced MSU-induced pain-like behavior triggered by mechanical stimulus and joint swelling (Fig. 1C,D).

### 15d-PGJ_2_-loaded NC reduce MSU-induced leukocyte migration to the knee joint and synovitis in a PPAR-γ-sensitive manner

**Figure 2:**
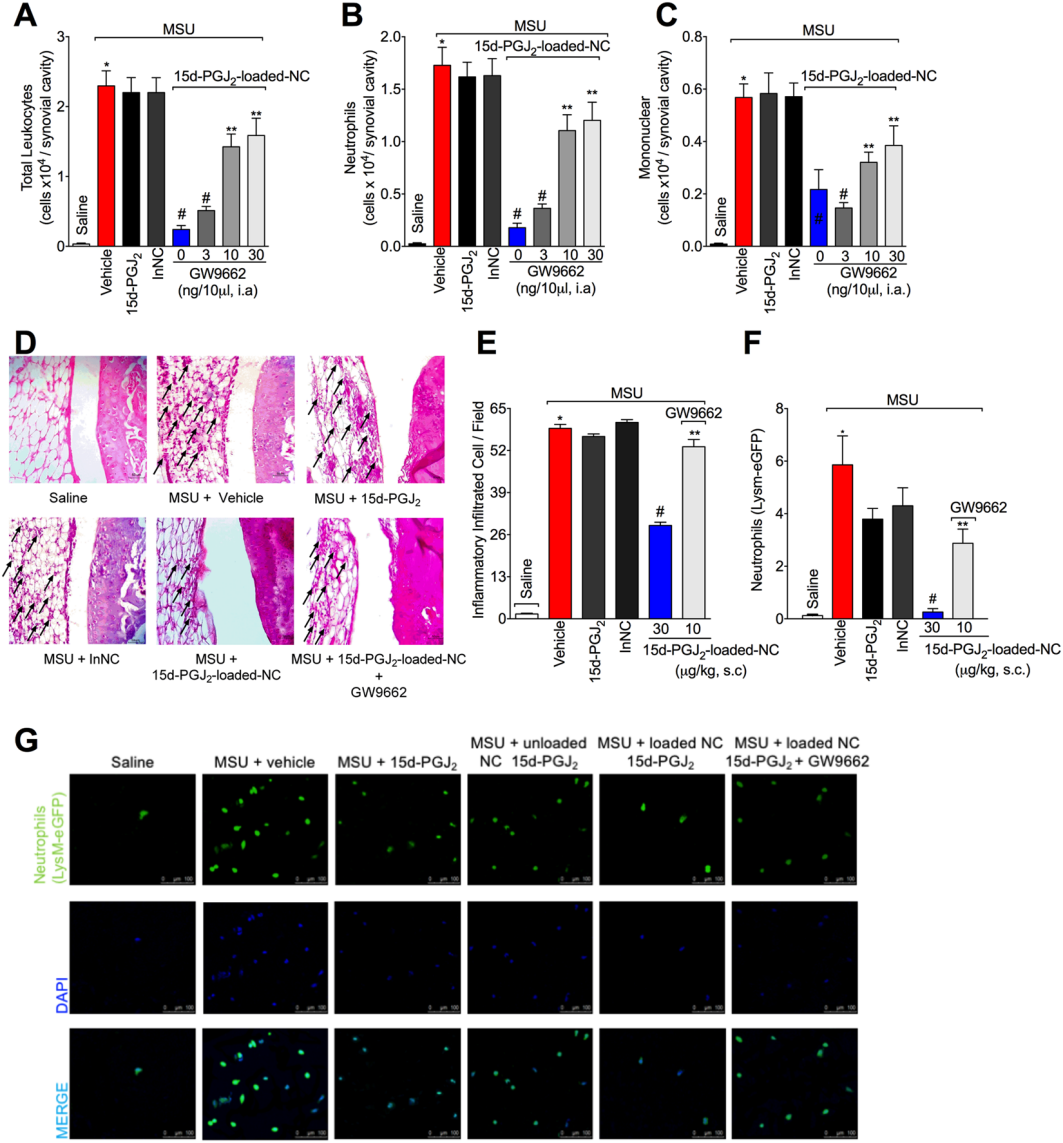
15d-PGJ_2_-loaded NC reduce MSU-induced leukocyte recruitment and synovitis in a PPAR-γ-sensitive manner. Fifteen hours after MSU, knee joints were collected for: counting of total leukocytes (**A**), neutrophils (**B**), mononuclear cells (**C**), Histopathological analysis by HE staining to asses synovitis (**D**) (Original magnification 400x) by a total score of inflammatory cells per field (**E**), and determination of LysM-GFP^+^ neutrophil recruitment by confocal microscopy (**F**,**G**). Original magnification 200x. Panel F shows the percentagem of LysM-eGFP + fluorescence. Results are mean ± SEM, n = 6, two independent experiments (*p < 0.05 vs. control group; ^#^p < 0.05 vs. vehicle mg/kg group, **p < 0.05 vs 15d-PGJ_2_-loaded NC. One-way ANOVA and post-test of Tukey).

Leukocyte migration to the inflamed foci, specifically neutrophils, is one hallmark of rheumatic diseases^2^. Thus, it was next addressed the effect of 15d-PGJ_2_-loaded NC in MSU-induced leukocyte recruitment and synovitis. Treatment with 15d-PGJ_2_-loaded NC reduced MSU-induced recruitment of total leukocyte (Fig. 2A), neutrophil (Fig. 2B), and mononuclear cells (Fig. 2C). Furthermore, 15d-PGJ_2_-loaded NC also reduced inflammatory infiltrate as observed in histopathological analysis as indicative of synovitis (Fig. 2D,E). Using MSU-stimulated LysM-eGFP+ mice, we observed that 15d-PGJ_2_-loaded NC reduced neutrophil recruitment to the knee joint, as observed by reduction in the fluorescence intensity by confocal microscopy (Fig. 2F,G). These effects produced by 15d-PGJ_2_-loaded NC were reverted by GW9662, as observed by an increase in MSU-induced of total leukocyte (Fig. 2A), neutrophil (Fig. 2B), mononuclear cells (2C), synovitis (Fig. 2D), leukocyte infiltration HE score analysis (Fig. 2E) and recruitment of LysM-GFP^+^ cells (Fig. 2F,G). These data indicate the involvement of PPAR-γ in 15d-PGJ_2_-loaded NC inhibitory effect over MSU-induced leukocyte recruitment. Neither free 15d-PGJ_2_ (without nanocapsule, at 30 μg/kg – the same dose of 15d-PGJ_2_-loaded NC) nor inert nanocapsules reduced MSU-induced leukocyte recruitment, synovitis and neutrophil recruitment (Fig. 2), indicating that nanoencapsulation improved the effect of 15d-PGJ_2_. 15d-PGJ_2_-loaded NC also reduced total leukocyte (SF1A), neutrophil (SF1B) and mononuclear cell (SF1C) counts 7 h after MSU injection. These data suggest that the effect of 15d-PGJ_2_-loaded NC may depend on inhibiting this inflammatory response and not only on speeding the inflammation resolution. We also tested a 10-fold higher dose of free 15d-PGJ_2_ (300 μg/kg, s.c., 30 min before MSU injection), which reduced MSU-induced mechanical hyperalgesia (SF2A), joint swelling (SF2B), and total leukocyte (SF2C), neutrophil (SF2D) and mononuclear cell (SF2E) counts. Therefore, reducing MSU-induced inflammation and pain is a pharmacological activity of 15d-PGJ_2_, however, it is necessary a higher dose of 15d-PGJ_2_ than 15d-PGJ_2_-loaded NC to achieve similar effects.

### 15d-PGJ_2_-loaded NC control the oxidative stress induced by MSU in a PPAR-γ-dependent manner

**Figure 3:**
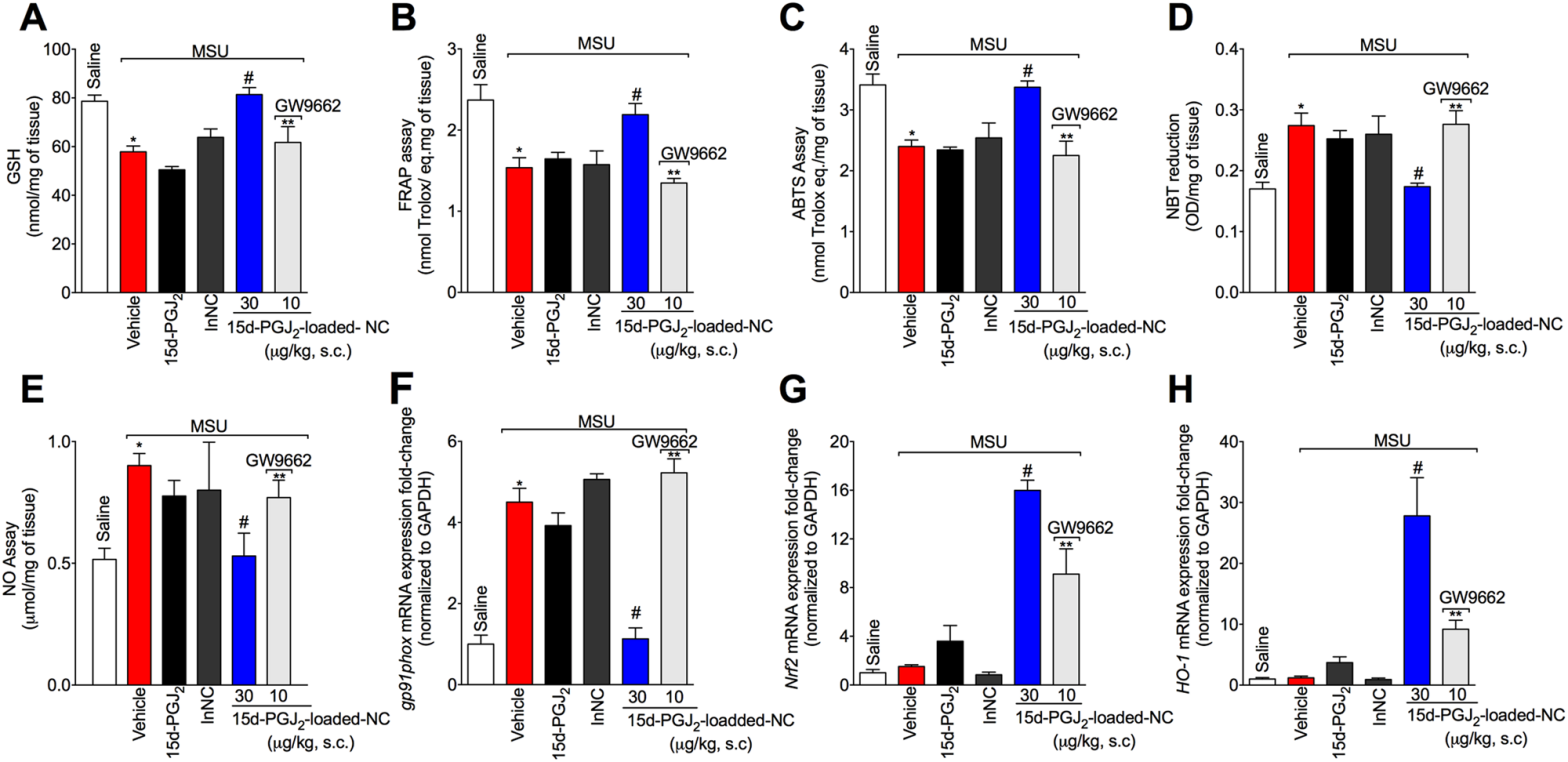
15d-PGJ_2_-loaded NC inhibit the oxidative stress induced by MSU in a PPAR-γ-dependent manner. Fifteen hours after MSU, knee joint was collected for the determination of oxidative stress by measuring GSH levels (**A**), FRAP assay (**B**), ABTS assay (**C**), superoxide anion prodution (NBT assay) (**D**), NO production (NO_2_^−^ assay) (**E**), and determination of the mRNA expression for *gp91phox* (**F**), *Nrf2* (**G**), and *Ho-1* (**H**) by RT-qPCR. Results are mean ± SEM, n = 6 mice per group in each experiment, two independent experiments (*p < 0.05 vs. control group; ^#^p < 0.05 vs. vehicle mg/kg group, **p < 0.05 vs 15d-PGJ_2_-loaded NC. One-way ANOVA and post-test of Tukey).

Given MSU crystals induce reactive oxygen and nitrogen species production^26,27^, it was next addressed whether 15d-PGJ_2_-loaded NC could reduce MSU-induced oxidative stress. MSU injection reduced GSH levels (Fig. 3A) and total antioxidant capacity [FRAP (Fig. 3B) and ABTS (Fig. 3C) assays] as well as increased superoxide anion (Fig. 3D) and NO production (Fig. 3E), and *gp91phox* mRNA expression (Fig. 3F). Treatment with 15d-PGJ_2_-loaded NC inhibited the MSU effects over parameters of Fig. 3A–F. The NADPH oxidase subunit gp91phox is required to superoxide anion production^28^, which reinforces the rationale of the results of Fig. 3.

Activation of PPAR-γ by different molecules results in increase of Nrf2/HO-1 signaling and thereby increase in the antioxidant defense^29–31^. Thus, we next investigated the effect of 15d-PGJ_2_-loaded NC in the Nrf2/HO-1 signaling. Treatment with 15d-PGJ_2_-loaded NC increased both *Nrf2* (Fig. 3G) and *HO-1* mRNA expression (Fig. 3H). These effects produced by 15d-PGJ_2_-loaded NC were reverted by GW9662, as observed by reduction in MSU-induced oxidative stress (Fig. 3), indicating the involvement of PPAR-γ. Neither free 15d-PGJ_2_ (without nanocapsule, at 30 μg/kg – the same dose of 15d-PGJ_2_-loaded NC) nor inert nanocapsules inhibited oxidative stress (Fig. 3), further indicating that nanoencapsulation improved the effect of 15d-PGJ_2_.

### 15d-PGJ_2_-loaded NC reduce MSU-induced pro-inflammatory cytokine production in a PPAR-γ-sensitive manner

**Figure 4:**
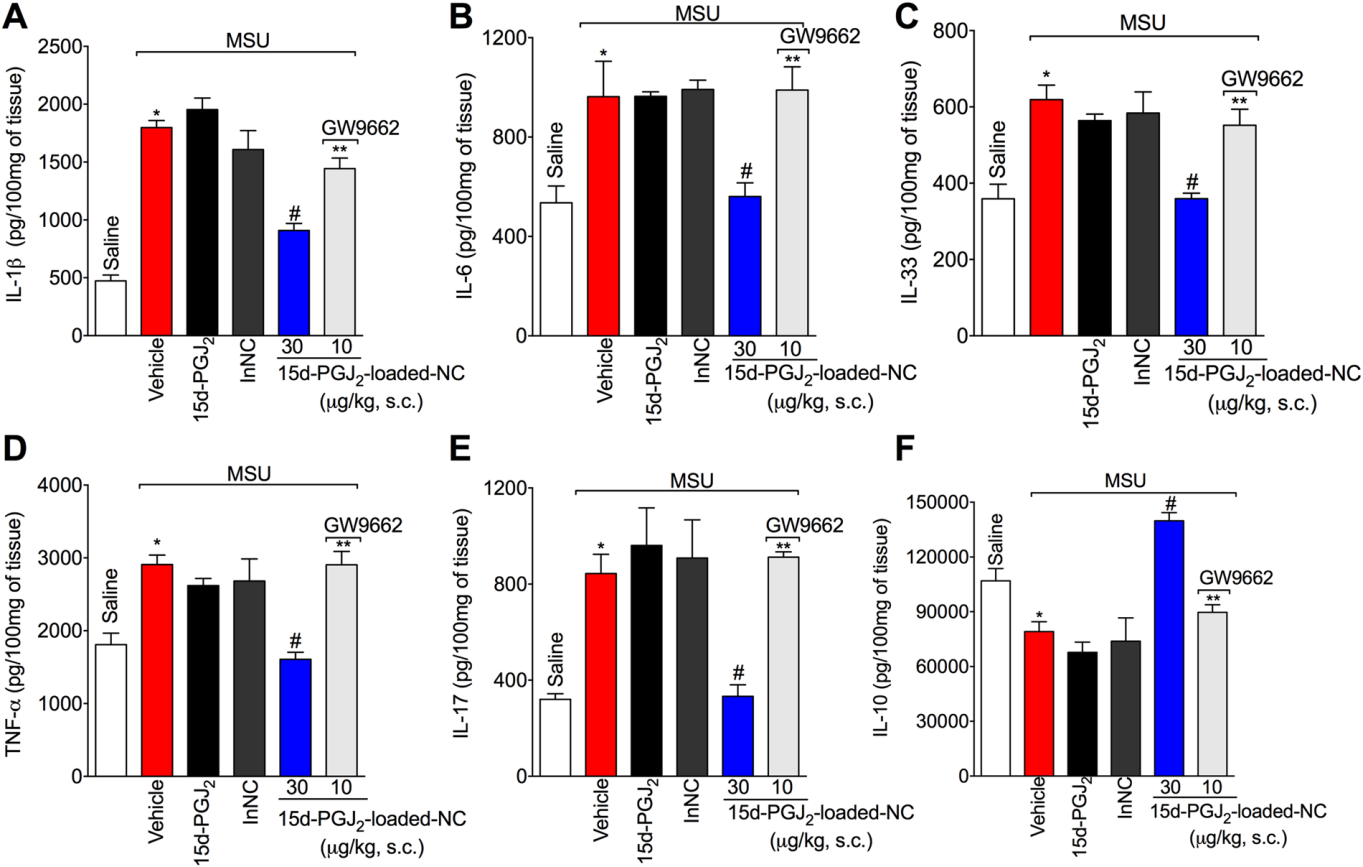
15d-PGJ_2_-loaded NC decrease inhibit MSU-induced cytokine production *in vivo* in a PPAR-γ-sensitive manner. Fifteen hours after MSU, knee joint was collected to quantitate IL-1β (**A**), IL-6 (**B**), IL-33 (**C**), TNF-α (**D**), IL-17 (**E**), and IL-10 (**F**) production by ELISA. Results are mean ± SEM, n = 6 mice per group in each experiment, two independent experiments (*p < 0.05 vs. control group; ^#^p < 0.05 vs. vehicle mg/kg group, **p < 0.05 vs 15d-PGJ_2_-loaded NC. One-way ANOVA and post-test of Tukey).

The next step was to investigate the effect of 15d-PGJ_2_-loaded nanocapsules on cytokine production and whether this effect was PPAR-γ-sensitive. Treatment with 15d-PGJ_2_-loaded NC decreased the levels of IL-1β (Fig. 4A), IL-6 (Fig. 4B), IL-33 (Fig. 4C), TNF-α (Fig. 4D), IL-17 (Fig. 4E), IL-10 (Fig. 4F). These effects produced by 15d-PGJ_2_-loaded NC were reverted by GW 9662, as observed by an increase in these same cytokines (Fig. 4), indicating that the 15d-PGJ_2_-loaded NC depends on PPAR-γ. Neither free 15d-PGJ_2_ (without nanocapsule, at 30 μg/kg – the same dose of 15d-PGJ_2_-loaded NC) nor inert nanocapsules reduced MSU-induced IL-1β (Fig. 4A), IL-6 (Fig. 4B), IL-33 (Fig. 4C), TNF-α (Fig. 4D), IL-17 (Fig. 4E), and IL-10 (Fig. 4F).

### 15d-PGJ_2_-loaded NC reduce MSU-induced IL-1β release in a PPAR-γ-sensitive manner

**Figure 5:**
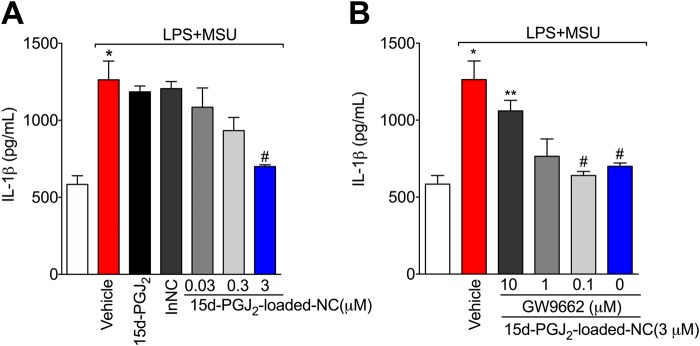
15d-PGJ_2_-loaded NC reduce MSU-induced IL-1β maturation *in vitro* in a PPAR-γ-sensitive manner. LPS-primed BMDMs were treated with 15d-PGJ_2_-loaded NC 0.03, 0.3, or 3 μM; or with free 15d-PGJ_2_ (**A**), or with 15d-PGJ_2_-loaded NC + GW9662 (10, 1 and 0.1 μm) (**B**) 30 min before MSU stimulation. Supernatants were collected 5 h after MSU stimulation and IL-1β levels were quantitated by ELISA. Results are mean ± SEM, n = 6 wells per group in each experiment, two independent experiments (*p < 0.05 vs. control group; ^#^p < 0.05 vs. vehicle mg/kg group, **p < 0.05 vs 15d-PGJ_2_-loaded NC. One-way ANOVA and post-test of Tukey).

The maturation of IL-1β is one of the main mechanisms of gout pathology^5,6^. To investigate whether 15d-PGJ_2_-loaded NC could reduce IL-1β release, an *in vitro* system with LPS-primed BMDM and stimulation with MSU crystals was used. IL-1β was measured in the supernatant of cells, indicating that it was released in its mature form^26^. First, it was performed concentration-response curve to select the optimal concentration of 15d-PGJ2-loaded NC. Only 15d-PGJ2-loaded nanocapsules at 3 μM reduced IL-1β maturation Fig. 5A, this concentration of 3 μM reduced 93% of IL-1β maturation. Next, a concentration-response curve of GW9662 was performed and we observed that the effect of 15d-PGJ_2_-loaded NC at 3 μM was inhibited only by GW9662 at 10 μM (Fig. 5B). The concentration of 10 μM reverted 80.3% of the protective effect of 15d-PGJ2-loaded NC. Neither free 15d-PGJ_2_ (without nanocapsule, at 3 μM – the same concentration of 15d-PGJ_2_-loaded NC) nor inert nanocapsules reduced IL-1β maturation (Fig. 5A)_._ None of the concentration used in this study reduced cell viability as per LDH and Trypan blue assays (data not shown). SF3 shows control data in which BMDMs from caspase-1/11 deficient mouse did not release IL-1β in the culture supernatant upon MSU stimulus compared to WT BMDMs.

### 15d-PGJ_2_-loaded NC reduce MSU-induced mRNA expression of inflammasome components and NF-κB activation in a PPAR-γ-sensitive manner

**Figure 6:**
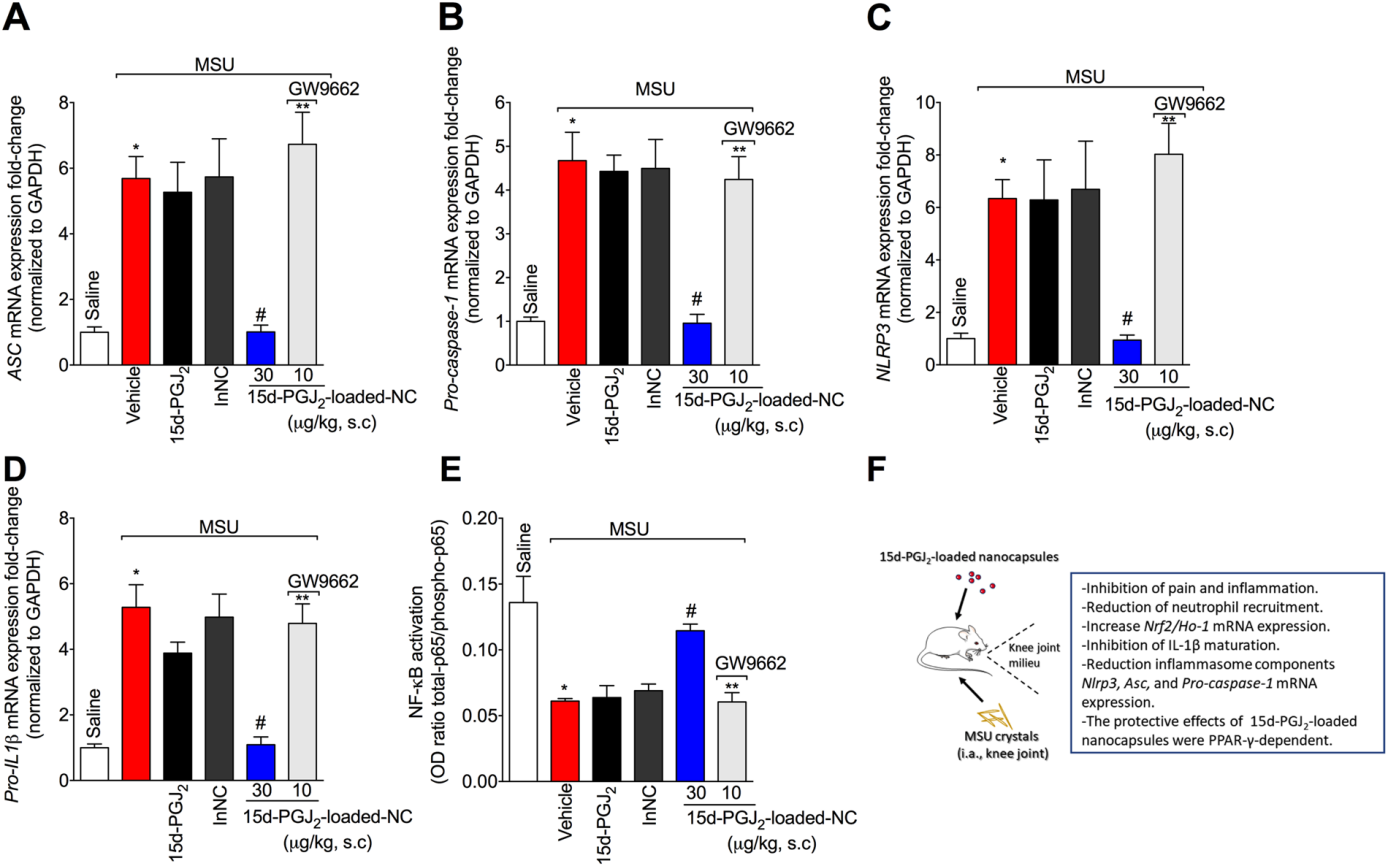
15d-PGJ_2_-loaded NC decrease MSU-induced *Nlrp3, Asc, Pro-caspase-1*, and *Pro-Il-1β* mRNA expression, and NF-κB activation in a PPAR-γ-sensitive manner. Fifteen hours after MSU, knee joint was collected for RT-qPCR assay to ascertain the mRNA expression of *ASC* (**A**), *Pro-caspase-1* (**B**), *NLRP3* (**C**), *Pro-Il-1β* (**D**), and ELISA to ascertain NF-κB activation (**E**). Schematic summary of data (**F**). Results are mean ± SEM, n = 6 mice per group in each experiment, two independent experiments (*p < 0.05 vs. control group; ^#^p < 0.05 vs. vehicle mg/kg group, **p < 0.05 vs 15d-PGJ_2_-loaded NC. One-way ANOVA and post-test of Tukey).

Given 15d-PGJ_2_-loaded nanocapsules reduced the production of IL-1β both *in vivo* and *in vitro*, it was investigated whether this molecule could reduce inflammasome components mRNA expression. Treatment with 15d-PGJ_2_-loaded NC diminished MSU-induced *ASC* (Fig. 6A), *Pro-caspase-1* (Fig. 6B), and *NLRP3* (Fig. 6C) mRNA expression, and also the *Pro-Il-1β* mRNA expression (Fig. 6D). Furthermore, 15d-PGJ_2_-loaded NC inhibited NF-κB activation as observed by the reduction in total-p65/phosphorylated-p65 OD ratio (Fig. 6E). The decrease in the ratio is attributed to the increase in the p65 subunit phosphorylated, and therefore, indicating activation (phosphorylation) of the NF-κB signaling pathway. The 15d-PGJ_2_-loaded NC inhibition of NF-κB activation lines up with the inhibition of inflammasome components mRNA expression. These effects produced by 15d-PGJ_2_-loaded NC were reverted by GW9662, as observed by an increase in the mRNA expression of the inflammasome components and NF-κB activation, indicating involvement of PPAR-γ in 15d-PGJ_2_-loaded NC effects. Neither free 15d-PGJ_2_ (without nanocapsule, at 30 μg/kg – the same dose of 15d-PGJ_2_-loaded NC) nor inert nanocapsules showed effect in these parameters (Fig. 6).

## Discussion

In this work, we show that 15d-PGJ_2_-loaded nanocapsules (NC) ameliorate MSU-induced pain and inflammation by reducing disease parameters such as leukocyte recruitment to the knee joint, oxidative stress, and pro-inflammatory cytokine production. Treatment with GW9662, a selective and irreversible inhibitor of PPAR-γ, reverted the benefic effect of 15d-PGJ_2_-loaded NC. Thus, indicating that the effect of 15d-PGJ_2_-loaded NC occurs in a PPAR-γ-sensitive manner. Moreover, the nanoencapsulation of 15d-PGJ_2_ improved its effect, given free 15d-PGJ_2_ at same dose (30 μg/kg) failed at inhibiting all parameters.

Activation of PPAR-γ has been shown to induce analgesia and to drive active resolution of inflammation in varied experimental models^14,15,24,32,33^. Topical treatment with 15d-PGJ_2_ reduces temporomandibular joint pain in rats^16^. Mechanistically, 15d-PGJ_2_ activates PPAR-γ that induces the hyperpolarization of nociceptor neurons by activating the NO/cGMP/K^+^ ATP channels signaling pathway through the release of β-endorphin^34,35^. In fact, activation of this signaling pathway is a mechanism by which morphine^36^, dypirone^37^, nitroxyl donor Angeli’s salt^38^, and some flavonoids such as diosmin^39^ and naringenin^40^ produce analgesic effect. Thus, this mechanism contributes to the analgesic properties of 15d-PGJ_2_. In our work, 15d-PGJ_2_-loaded NC was used. The nanoencapsulation of 15d-PGJ_2_ results in a reduction of up to 33 times in the active dose of 15d-PGJ_2_ compared to its non-nanoencapusaled form (30 μg/kg *vs*. 1000 μg/kg) to reduce inflammation^20^ and pain^41^. Herein, we show that free 15d-PGJ_2_ at 30 μg/kg did not present analgesic effect. Considering nanoencapsulation of 15d-PGJ_2_ promoted an analgesic effect with a dose that is inactive when not nanoencapsulated, it conceivable that the process of nanoencapsulation improves the efficacy of 15d-PGJ_2_.

Oxidative stress contributes to the development and maintenance of both peripheral and central sensitization^42^. Thus, molecules that target (directly or indirectly) oxidative stress are interesting therapeutic approaches as analgesic molecules^42^. Intrathecal administration of a ROS donor increases the excitability of nociceptors in the dorsal horn of the spinal cord^43^ and intraplantar or intraperitoneal injection of ROS donors is sufficient to induce pain-like behaviors in naïve animals^44–47^. Concerning gout, promising analgesic and anti-inflammatory activities in MSU-induced arthritis were demonstrated for antioxidant molecules^26,48,49^. Furthermore, ROS also mediate neutrophil recruitment^50^ *via* ROS-dependent glutathionylation of neutrophils’ actin^51^. In turn, neutrophils produce ROS upon recognition of MSU crystals^52^. Herein, we show that 15d-PGJ_2_-loaded NC reduced MSU-induced oxidative stress. Part of the pro-resolving effects of 15d-PGJ_2_ is related to its antioxidant activity^7^. In fact, 15d-PGJ_2_ induces Nrf2 activation and increases the expression of the scavenger receptor CD36 in murine macrophages^9^. Activation of PPAR-γ by varied molecules up-regulates Nrf2/HO-1 signaling and thereby increase the endogenous antioxidant defenses^29–31^. Therefore, the increase of *Nrf2*/*Ho-1* mRNA expression might account to the analgesic and anti-inflammatory activity of 15d-PGJ_2_-loaded NC.

The maturation of IL-1β is a crucial step in the pathogenesis of gout^4–6^. A study with 448 Chinese patients with recurrent gout shows that macrophages, peripheral blood mononuclear cells (PBMCs), and synoviocytes from these patients with a variant allele that reduce PPAR-γ activity present increased NLRP3 activity and IL-1β release^53^. A siRNA targeting this PPAR-γ allele variant also increases NLRP3 mRNA expression^53^, indicating that PPAR-γ limits MSU-induced inflammation. In MSU-induced inflammation and pain, NLRP3 inflammasome assembly is fundamental to IL-1β maturation^5,6^. In the present study, we show that 15d-PGJ_2_-loaded NC reduced MSU-induced IL-1β release in BMDM culture supernatant in a PPAR-γ-sensitive manner since the treatment with GW9662 reverted the 15d-PGJ_2_-loaded NC activity. Our data corroborate other studies showing that 15d-PGJ_2_^54^ or pioglitazone (other PPAR-γ activator)^55^ reduce MSU-induced pro-inflammatory cytokine production. In comparison to the study of Akahoshi and collaborators^54^, we used a concentration 16 times lower (3 μM *vs*. 50 μM) indicating that nanoencapsulation increased 15d-PGJ_2_ efficacy compared to the non-nanoencapsulated form. Treatment with 15d-PGJ_2_-loaded NC reduced the MSU-induced production of other pro-inflammatory cytokines such as TNF-α, IL-17, and IL-33. This is important given IL-1β^5^, TNF-α^56^, IL-17^57^, and IL-33^58,59^ mediate neutrophil recruitment in rheumatic disease. In fact, using three different methodologies (leukocytes count in Neubauer chamber, HE staining, and MSU injection in LysM-eGFP mice), we show that PGJ_2_-loaded NC reduced neutrophil and mononuclear cell recruitment. Recruited neutrophil produce IL-1β, TNF-α, endothelin-1, and PGE_2_ that altogether either sensitize nociceptor neurons or activate them^2,60,61^. Furthermore, MSU crystals induce the release of NETs by neutrophils^62^, which is increased by IL-1β^62,63^. Thus, inhibition of pro-inflammatory cytokine production, especially IL-1β (*in vivo* and *in vitro*), is fundamental to the analgesic effect of 15d-PGJ_2_-loaded NC. Moreover, we show 15d-PGJ_2_-loaded NC reduced NF-κB activation *in vivo*, corroborating other study that demonstrates 15d-PGJ_2_ reduces NF-κB activation in a model of asthma^64^. Mechanistically, activation of PPAR-γ by 15d-PGJ_2_ inhibits the transcriptional response mediated by AP-1 and NF-κB in macrophages^10^. This was observed through the reduction in the luciferase activity of TATA-containing promoter linked to these transcription factors^10^. Furthermore, 15d-PGJ_2_ also reduces NF-κB activation by direct mechanism, *i.e*., that does not depend on PPAR-γ activation. This mechanism is related to covalently binding to IKK^12^ or to the alkylation of the cysteine residue Cys38 of the NF-κB subunit p65^11^. Activation of NF-κB drives the production of pro-inflammatory mediators and components of inflammasome^65^. Thus, inhibition of this transcription factor certainly contributed to the analgesic and anti-inflammatory effects of 15d-PGJ_2_-loaded NC.

To conclude, in this work we show that nanoencapsulation of 15d-PGJ_2_ increased the effect of this molecule, given free 15d-PGJ_2_ (*i.e*. the same dose and non nanocapsulated) failed at inhibiting MSU-induced pain and inflammation. The protective effect of 15d-PGJ_2_-loaded NC is related to the inhibition of MSU-induced oxidative stress as observed by the increase of antioxidant defense and *Nrf2/Ho-1* mRNA expression. In addition, treatment with 15d-PGJ_2_-loaded NC reduced MSU-induced pro-inflammatory cytokine production, especially IL-1β (both *in vivo* and *in vitro*) and neutrophil recruitment by a mechanism involving the reduction of NF-κB activation in the context of MSU inflammation. Moreover, the 15d-PGJ_2_-loaded NC effects were PPAR-γ-sensitive, given they were amenable by GW 9662 treatment. Thus, herein, we show that 15d-PGJ_2_-loaded NC possesses protective effect in MSU-induced pain and inflammation and this protective effect is PPAR-γ-sensitive. These results were summarized in Fig. 6F.

## Materials and Methods

### Experimental procedures

Mice were treated s.c. with 15d-PGJ_2_-loaded NC at the doses of 3, 10, or 30 μg/kg 30 min before MSU injection. The 15d-PGJ_2_-loaded NC effect on pain-like behavior triggered by mechanical stimulus and joint swelling induced by MSU crystals were determined and the dose of 30 μg/kg was selected for the subsequent experiments. Next, mice were treated with GW9662 at the doses of 3, 10, or 30 ng (i.art., 30 min before MSU injection) to determine if this PPARγ receptor antagonist would inhibit the analgesic and anti-inflammatory effects of 15d-PGJ_2_-loaded NC. GW9662 at the dose of 10 ng was selected for the subsequent experiments. In all experiments, mice were treated with 15d-PGJ_2_-loaded NC, inert nanocapsules (InNC), free 15d-PGJ_2_ (without nanocapsules), or 15d-PGJ_2_-loaded NC+ GW9662. All analyzes were conducted 15 h after MSU injection. Leukocyte recruitment was evaluated in the knee joint wash by Neubauer chamber and Rosenfelt stained slices (total leukocyte, neutrophil, and mononuclear cells count) and in MSU-stimulated LysM-eGFP+ mice by confocal microscopy. Histopathological analysis in the knee joint was assessed by HE staining. MSU-induced oxidative stress in the knee joint was evaluated by measuring GSH levels, total tissue antioxidant activity (FRAP and ABTS assays), and production of superoxide anion and nitrite. Further addressing oxidative stress, *gp91phox*, *Nrf2*, and *Ho-1* mRNA expression were evaluated by RT-qPCR. MSU-induced pro-inflammatory cytokine production was assessed *in vivo* by ELISA in the knee joint. The mRNA expression of *Nlrp3* inflammasome components in the knee joint was determined by RT-qPCR. The maturation of IL-1β was determined by ELISA in the supernatant of LPS-primed BMDM and stimulated with MSU crystals. NF-κB activation in the knee joint was also determined by ELISA. The 15d-PGJ_2_-loaded NC and InNC were prepared and characterized as previously described^20^. The 15d-PGJ_2_ (non-nanocapsulated) and GW9662 were acquired from Sigma (Sigma Chemical Co., St. Louis, MO, USA). Supplemental Figures’ (SF) experiments were conducted as mentioned above except that leukocyte recruitment was determined 7 h after MSU injection (SF1), a higher dose of 15d-PGJ_2_ (300 μg/kg) was also tested in MSU arthritis (SF2), and the effect of caspase-1/11 deficiency over IL-1β release in BMDMs culture supernatant was verified (SF3).

### Preparation of the PLGA nanocapsules with 15d-PGJ_2_

As described by Alves *et al*.^20^, the poly (d,l-lactic-co-glycolic acid, 50:50) (PLGA) nanocapsules were prepared by a nanoprecipitation method^66^ by mixing an organic phase into an aqueous phase. The organic phase consisted of PLGA polymer (100 mg), acetone (30 mL), 15d-PGJ_2_ (100 mg), sorbitan monostereate (40 mg) and caprylic/capric acid triglyceride (200 mg). The aqueous phase consisted of polysorbate 80 (60 mg) and deionized water (30 mL). After disintegration of the components of both phases, the organic phase was gradually added to the aqueous phase, and the suspension agitated during 10 min. The suspension was concentrated to a volume of 10 mL under low pressure using a rotary evaporator to achieve a suspension of 15d-PGJ_2_ with a final concentration of 10 µg/mL. A control formulation (without 15d-PGJ_2_) was also prepared, following the same procedures. Zeta potential measurement and efficiency of association of 15d-PGJ_2_ in the PLGA nanocapsules were used to ensure parameters such size and polydispersion measurements. Parameters of morphology and structure of PLGA nanocapsules with 15d-PGJ_2_ were also assessed in a JEOL 1200EX II microscope, a Transmission electron microscopy (TEM) operating at 80 kV. A Nanosurf Easy Scan 2 Basic atomic force microscope (BT02217, Nanosurf, Switzerland) was used to verify the diameter of PLGA nanoparticles in suspension and size distribution by using the Nanosurf software^20,41^.

### Animals

Male Swiss mice (25–30 g) from the Universidade Estadual de Londrina, Londrina, Paraná, Brazil, and WT C57BL/6 background and caspase-1/11 deficient C57BL/6 background mice from Ribeirao Preto Medical School, University of São Paulo were used in this study. Mice were housed in standard clear plastic cages with free access to food and water with a light/dark cycle of 12/12 h at a constant temperature of 21° +/− 1 °C. All behavioral testing was performed in a temperature-controlled room (21° + /− 1 °C) between 9 a.m. and 5 p.m. Animal care and handling procedures were in accordance with the International Association for Study of Pain guidelines, and the Ethics Committee of the Universidade Estadual de Londrina approved all procedures of this study (process number 14600.2013.73). All efforts were made to minimize animal suffering and to reduce the number of animals used.

### Induction of MSU-induced knee joint inflammation

Joint inflammation was induced by the intra-articular (i.a.) administration of MSU (100 μg/10 µL) into the right knee joint of mice under isoflurane anesthesia. Control animals received an i.a. injection of sterile saline (10 μL)^26^.

### Evaluation of knee joint hyperalgesia

Joint mechanical hyperalgesia was assessed using an electronic von Frey apparatus. Mice were accomodated in acrylic cages with a wire grid floor, and the stimulations were performed only when the animals were quiet (and with the four paws on the grid floor). This test consists of an electronic pressure-meter, with a force transducer fitted with polypropylene tip (Insight instruments, Ribeirao Preto, SP, Brazil). To assess the articular pain, a large tip (4.15 mm^2^) was used to exclude the cutaneous nociception^67^. A progressive perpendicular pressure was applied to the central area of the plantar surface of the hind paw to induce flexion of the femur-tibial joint followed by the hind paw withdrawal. The intensity of the force applied (in g) at the moment of paw withdrawal was automatically recorded. The test was performed at the times indicated on figures. The investigators were blinded to the treatment.

### Knee joint swelling evaluation

A caliper (Mitutoyo, IL, USA) was used to determine the knee joint swelling before (baseline), and after MSU injection (100 μg/10 µL, i.a.). Time points of evaluation were indicated in the figures. Knee joint swelling was determined for each mouse by the difference between the time point indicated on figures and the baseline. The joint swelling value is expressed as Δmm/joint.

### *In vivo* leukocyte migration

Leukocyte migration into the knee joint was assessed 15 h after i.a. injection of MSU crystals^59^. Articular cavities were washed with saline containing 1 mM EDTA (Sigma Chemical Co., St. Louis, MO, USA), and diluted to a final volume of 50 μL with PBS/EDTA. A Neubauer chamber was used to count the total number of leukocytes in samples diluted in Turk solution. Differential cell counts were performed in Rosenfeld stained slices to distinguish polymorphonuclear from mononuclear cells using a light microscope.

### Histopathological analysis

Knee joint specimens were collected 15 h after stimulus. Fixation was performed with 10% paraformaldehyde in PBS and decalcification was performed with EDTA (Sigma Chemical Co., St. Louis, MO, USA). Samples were embedded in paraffin and sectioned for histological analysis. Tissue sections were stained with hematoxylin and eosin for morphological evaluation. Results are expressed as the number of leukocytes per field using a magnification of ×400 and slice dimension of 569 × 633 pixels^26^.

### Fluorescence assay

Knee joint wash of LysM-GFP mice was collected in sterile slides 15 h after MSU injection into the knee joints. DAPI fluorescent stain (ThermoFisher, MA, USA) was added to slides for localization of nucleus in each sample. The representative images and quantitative analysis were performed using a confocal microscope (SP8, Leica Microsystems, Mannheim, Germany). The intensity of fluorescence was quantified in randomly selected fields of different groups by an investigator blinded to the treatments. Results are presented as the percentage of GFP fluorescent intensity.

### GSH levels measurement

Samples of articular joint were collected and stored at −80 °C for at least 48 h. The sample was homogenized with 200 μL of 0.02 M EDTA (Sigma Chemical Co., St. Louis, MO, USA). The homogenate was mixed with trichloroacetic acid 50% and and was homogenized three times over 15 min, and then, centrifuged (15 min × 1500 g × 4 °C). The supernatant was mixed with 200 μL of 0.2 M TRIS buffer, pH 8.2, and 10 μL of 0.01 M DTNB (Sigma Chemical Co., St. Louis, MO, USA) and let to react during 5 min. Sample was read at 412 nm (Multiskan GO Microplate Spectrophotometer, Thermo Scientific, Vantaa, Finland) against a blank control. A standard GSH curve allowed calculating the GSH levels per mg of tissue^26^.

### ABTS and FRAP assays

The tissue antioxidant properties were determined by their free radical scavenging (ABTS [2,2′-Azinobis-3-ethylbenzothiazoline 6-sulfonic acid] assay, Sigma Chemical Co., St. Louis, MO, USA) and ferric reducing (FRAP assay, Sigma Chemical Co., St. Louis, MO, USA) properties. The tests were adapted to a 96-well microplate format as previously described^26^. Articular tissue samples were collected 15 h after MSU i.a injection (100 μg/10 µL) and homogenized immediately in ice-cold KCl buffer (500 μL, 1.15% w/v). The homogenates were centrifuged (200 g × 10 min × 4 °C), and the supernatants were used in both assays. ABTS solution (200 µL) and 10 μL of sample were added to each well and let to react during 6 min incubated at 25 °C followed by reading at 730 nm. For FRAP assay, the supernatants (10 μL) were mixed with the freshly prepared FRAP reagent (150 μL) and let to react during 30 min at 37 °C followed by reading at 595 nm (Multiskan GO Microplate Spectrophotometer, Thermo Scientific, Vantaa, Finland). A standard Trolox curve (Sigma Chemical Co., St. Louis, MO, USA) was used in the ABTS and FRAP assays.

### Superoxide anion production

The nitroblue tetrazolium (NBT, Amresco, Solon, OH, USA) assay was adapted to a microplate to determine superoxide anion production as described previously^26^. Tissue homogenates were diluted in 1.15% KCl (10 mg/mL) (Sigma Chemical Co., St. Louis, MO, USA). The NBT reduction was read at 600 nm (Multiskan GO Microplate Spectrophotometer, Thermo Scientific, Vantaa, Finland). The tissue weight was used for data normalization.

### Nitrite production

Samples from knee joint were collected 15 h after MSU injection, homogenized in 500 μL of saline, and nitrite (NO_2_^−^) concentration was determined by the Griess reaction (Sigma Chemical Co., St. Louis, MO, USA) as an indicator of nitric oxide (NO) production^68^. Results are μmol of NO_2_^−^ per mg of tissue.

### Reverse transcription and quantitative polymerase chain reaction (RT-qPCR)

Samples (e.g. knee joints) were collected and homogenized in the TRIzol® reagent (ThermoFisher, MA, USA). Time point of collection was 15 h after MSU injection. Total RNA was extracted according to manufacturer’s directions. Reverse transcription of total RNA to cDNA and qPCR were performed using GoTaq® 2-Step RT-qPCR System (Promega Corporation, WI, USA) and specific primers (Applied Biosystems®, ThermoFisher, MA, USA). The mRNA level of glyceraldehyde 3-phosphate dehydrogenase (*Gapdh*) was used as reference gene.

### Preparation of bone marrow-derived macrophages (BMDMs) and inflammasome activation assay

Bone marrow cells were collected from femora and tibiae of C57BL/6 mice (8 weeks old) and cultured in RPMI 1640 medium (Sigma Chemical Co., St. Louis, MO, USA) containing 10% FBS (Sigma Chemical Co., St. Louis, MO, USA) and 15% L929 cell conditioned medium. After 7 days, BMDMs were plated at the density of 1.5 × 10^6^ cells/well in 96-well plate. BMDM were primed with lipopolysaccharide (LPS; 500 ng/mL) from *Escherichia coli* (Santa Cruz Biotechnology, TX, USA). After 3 h, BMDMs received 450 µg/mL of MSU to stimulate NLRP3 inflammasome-dependent IL-1β maturation^6^. BMDMs were treated with 15d-PGJ_2_-loaded NC at 0.03, 0.3, or 3 μM 30 min before MSU stimulation. To investigated the influence of PPAR-γ, BMDM were co-treated with 15d-PGJ_2_-loaded NC+ GW 9662 at 0.1, 1, or 10 μM. BMDMs were also treated with inert NC and free 15d-PGJ_2_ at the same concentration chosen for 15d-PGJ_2_-loaded NC (3 μM). Supernatants were also collected 5 h after MSU stimulation. Lactate dehydrogenase (LDH) release in the supernatant and Trypan blue assays were used as a marker of cellular viability.

### Cytokine measurement

Knee joint samples were homogenized in a buffer containing protease inhibitors (500 μL of 1 mM Phenylmethanesulfonyl fluoride, Sigma Chemical Co., St. Louis, MO, USA). IL-1β, TNF-α, IL-6, IL-17 and IL-33 levels were determined using eBioscience ELISA kits (eBioscience, San Diego, CA, USA). The results are picograms (pg) of cytokine/mg of tissue.

### NF-κB activation

Knee samples were collected in ice-cold lysis buffer (Cell Signaling), homogenized and centrifuged (16,100 *g* × 10 min × 4 °C). The resulting supernatants were used to assess the levels of total NF-κB p65 subunit and phosphorylated NF-κB p65 subunit by ELISA using PathScan kits #7836 and #7834, respectively (Cell Signaling Technology, Beverly, MA, USA). Results are OD ratio (total p65/phospho-p65) at 450 nm (Multiskan GO Microplate Spectrophotometer, Thermo Scientific, Vantaa, Finland).

### Data analysis

Data analyzes was performed using the Prism 6.0 statistical program (GraphPad software, Inc.). Comparison between groups and doses at all times when the parameters were measured at different time points after the stimulus injection was performed using two-way ANOVA. The analyzed factors were treatments, time, and time versus treatment interaction. For single time-point, we used one-way ANOVA and the post-test of Tukey. *P* < 0.05 was considered significant.

## Electronic supplementary material

Suplementary Figures

## Data Availability

All data are presented in the manuscript.
