# Supplementary material for: 15d-PGJ2-loaded nanocapsules ameliorate experimental gout arthritis by reducing pain and inflammation in a PPAR-gamma-sensitive manner in mice
Source: Sci Rep. 2018 Sep 18;8:13979. doi: 10.1038/s41598-018-32334-0 (PMC6143605; doi:10.1038/s41598-018-32334-0)
Supplement: Supplementary file 1 — Suplementary Figures [file 41598_2018_32334_MOESM1_ESM.pdf]

Suplemmentary Information

15d-PGJ_2-loaded nanocapsules ameliorate experimental gout arthritis by reducing pain and inflammation in a PPAR-gamma-sensitive manner in mice

Kenji W. Ruiz-Miyazawa[a], Larissa Staurengo-Ferrari[a], Felipe A. Pinho-Ribeiro[a], Victor Fattori[a], Tiago H. Zaninelli[a], Stephanie Badaro-Garcia[a], Sergio M. Borghi[a], Ketlem C. Andrade[a], Juliana T. Clemente-Napimoga[b], Jose C. Alves-Filho[c], Thiago M. Cunha[c], Leonardo F. Fraceto[d], Fernando Q. Cunha[c], Marcelo H. Napimoga[b], Rubia Casagrande[e], and Waldiceu A. Verri, Jr.[a,*]

[a]Departamento de Ciências Patológicas, Universidade Estadual de Londrina-UEL, Rod. Celso Garcia Cid, Km 380, PR445, 86057-970, Cx. Postal 10.011, Londrina, Paraná, Brazil.
[b]Laboratory of Immunology and Molecular Biology, São Leopoldo Mandic Institute and Researcher Center, Campinas, Brazil
[c]Department of Pharmacology, Ribeirão Preto Medical School, University of São Paulo, Avenida Bandeirantes s/n, 14050-490, Ribeirão Preto, São Paulo, Brazil
[d]Department of Environmental Engineering, São Paulo State University, Sorocaba, Brazil
[e]Departamento de Ciências Farmacêuticas, Universidade Estadual de Londrina-UEL, Avenida Robert Koch, 60, Hospital Universitário, 86038-350, Londrina, Paraná, Brazil.

*Author to whom correspondence should be addressed: Waldiceu A. Verri Jr. Departamento de Ciências Patológicas, Universidade Estadual de Londrina-UEL, Rod. Celso Garcia Cid, Km 380, PR445, 86057-970, Cx. Postal 10.011, Londrina, Paraná, Brazil. Fax: + 55 43 33714387, Tel: + 55 43 33714979, E-mail address: waldiceujr@yahoo.com.br; waverri@uel.br.

Supplementary Figures

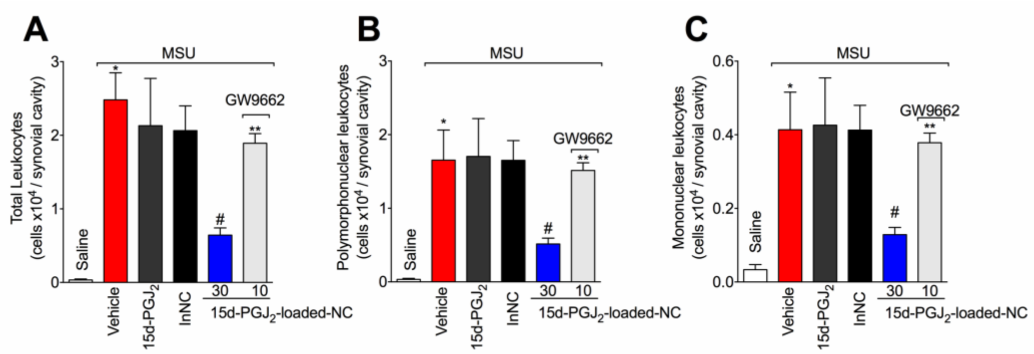

Supplementary Figure 1 (SF1). 15d-PGJ2-loaded NC reduce MSU-induced leukocyte recruitment 7h post-stimulus in a PPAR-γ-sensitive manner. Seven hours after MSU stimulus, the number of total leukocytes (**A**), neutrophils (**B**) and mononuclear cells (**C**) were determined. Results are expressed as mean ± SEM, n = 6, two independent experiments (*p < 0.05 vs. control group; #p < 0.05 vs. vehicle mg/kg group, **p<0.05 vs 15d-PGJ2-loaded NC).

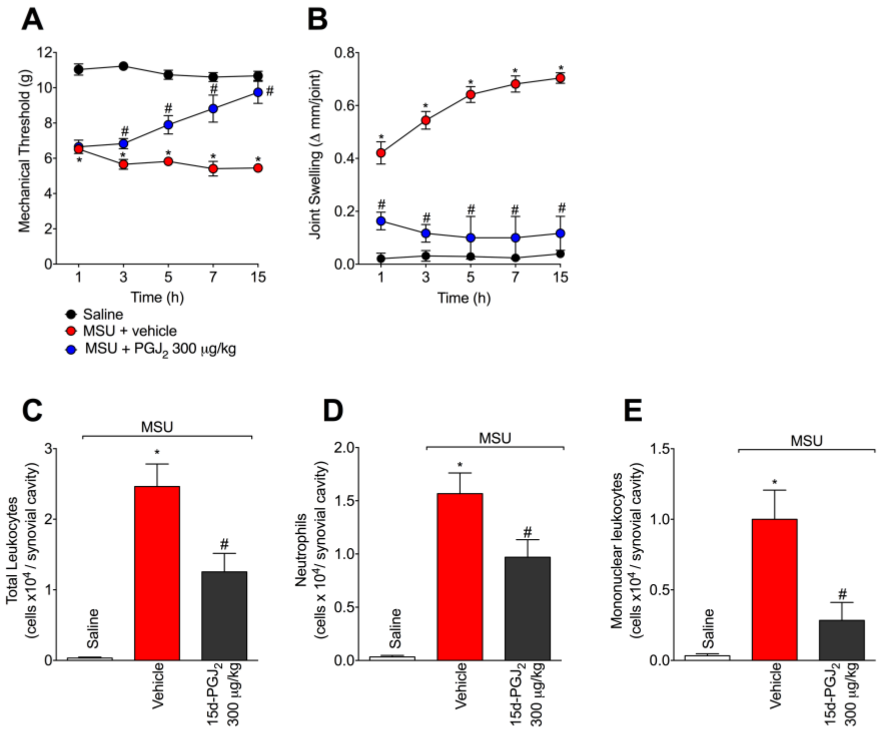

Supplementary Figure 2 (SF2). Higher dose of non-encapsulated 15d-PGJ$_2$ inhibits MSU-induced mechanical hyperalgesia and joint swelling. Mice were treated with 15d-PGJ$_2$ (300 µg/kg) or vehicle 30 minutes before MSU (100 µg/10 µl/knee) stimulus in the femur-tibial joint of mice. Mechanical hyperalgesia (**A**) and joint swelling (**B**) were evaluated 1, 3, 5, 7, and 15h after MSU injection and 15h after MSU injection knee joints were collected to determine total leukocytes (**C**), neutrophil (**D**) and mononuclear cell counts using Neubauer chamber and stained slices (*$p < 0.05$ vs. control group; #$p < 0.05$ vs. vehicle group two-way ANOVA followed by Tukey's post-test).

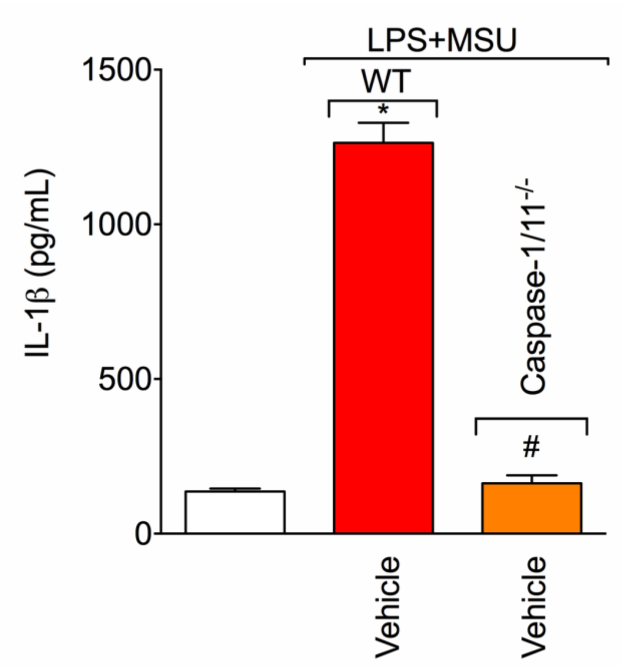

Supplementary Figure 3 (SF3). MSU-induced IL-1β maturation *in vitro* depends on caspase-1. IL-1β concentration (ELISA) in WT and caspase-1/11$^{-/-}$ BMDM culture supernatants primed with LPS (500 ng/ml, i.e. signal 1) for 3h and stimulated with MSU (450 μg/ml, *i.e.* signal 2) followed by 5h incubation. Results are expressed as mean ± SEM, n = 6 wells per group per experiment, two independent experiments (*$p < 0.05$ vs. control group; #$p < 0.05$ vs. WT vehicle group, one-way ANOVA followed by Tukey's post-test).
